# Retrobulbar triamcinolone for inflammatory choroidal neovascularization in pregnancy

**DOI:** 10.1186/s12886-020-01759-5

**Published:** 2020-12-09

**Authors:** Emilia Maggio, Maurizio Mete, Antonio Polito, Gloria Parrozzani, Grazia Pertile

**Affiliations:** IRCCS Sacro Cuore Don Calabria Hospital, Via Don Sempreboni 5 - Negrar, 37024 Verona, Italy

**Keywords:** Inflammatory choroidal neovascularization, Pregnancy, Retrobulbar triamcinolone injection

## Abstract

**Background:**

Choroidal neovascularization (CNV) in pregnancy has rarely been described. A differential diagnosis between inflammatory, idiopathic, and myopic CNV may be challenging. Moreover, there is no consensus on management, and therapeutic options may be further limited by patient and physician concerns about potential risk to the fetus. Herein, we report a case of inflammatory CNV during pregnancy and describe a previously unreported management approach with retrobulbar triamcinolone injections.

**Case presentation:**

A 36-year-old woman presented with vision loss and metamorphopsia in her right eye while 21 weeks pregnant. She was diagnosed with an inflammatory CNV based on the following multimodal imaging findings: a type 2 lesion with the “pitchfork sign” on OCT, along with the absence of tomographic signs of myopic CNV, and the presence on autofluorescence of multiple hyper-autofluorescent spots, interpreted as focal areas of inflammation at the level of the outer retina and inner choroid. The patient refused oral corticosteroids and any intravitreal injection therapies. Therefore, she was treated with two trans-Tenon’s retrobulbar injections of triamcinolone acetonide after explaining the procedure and acquiring consent. The treatment resulted in a regression of inflammatory signs and a reduction of neovascular activity. No adverse events occurred for the mother or the baby, neither during the pregnancy nor after delivery.

**Conclusion:**

Inflammatory CNV may be rarely associated with pregnancy. The correct diagnosis is crucial to allow the consideration of all possible management options. To the best of our knowledge, this is the first reported case of treatment with retrobulbar triamcinolone injections. This may represent a suitable therapeutic option in the absence of any other therapeutic approaches.

## Background

Choroidal neovascularization (CNV) may represent a vision-threatening complication of inflammatory eye diseases [1, 2]. It is thought to be caused by a local angiogenic stimulus related to inflammation, or result from a degenerative disruption in the retinal pigment epithelium (RPE)-Bruch’s membrane complex [2]. Inflammatory CNV has been described in a variety of inflammatory eye disorders, including infectious and non-infectious uveitis, serpiginous choroiditis, presumed ocular histoplasmosis syndrome, Vogt-Koyanagi-Harada disease, and White-Dot Syndromes (WDS) such as multifocal choroiditis, punctate inner choroiditis, and multiple evanescent white dot syndrome [2, 3]. It has rarely been described in association with pregnancy, either in conjunction or not with the aforementioned inflammatory conditions [4–6].

In young myopic pregnant women, a differential diagnosis between inflammatory CNV and idiopathic or myopic CNV may be challenging. Indeed, some cases of idiopathic CNV in pregnancy have also been reported, probably related to an increase in angiogenic factor activity during pregnancy [4, 7].

Moreover, CNV management in pregnancy is poorly elucidated. Previously, several treatment options have been safely adopted in pregnant women [6–10]. In particular, the use of intravitreal anti-VEGF injections [6–10] and triamcinolone in pregnancy, for both sub-Tenon’s and intravitreal administration [6–10], has been described. However, although most cases have not shown any harm for the mother or the baby, it is not possible to drawn definitive conclusions on their safety, given the scarcity of published cases. In addition, patients may express concerns regarding consent for treating these conditions to avoid any potential risk to the fetus. This further limits the therapeutic possibilities.

Herein, we report a rare case of inflammatory CNV in pregnancy. Moreover, we describe the previously unreported management of such a condition with retrobulbar triamcinolone injections, performed because the patient refused any other intravitreal or oral treatment.

## Case presentation

A 36-year-old Caucasian woman presented with vision loss and metamorphopsia in her right eye. She was 21 weeks pregnant and reported a previous uncomplicated pregnancy that progressed to full term. Her prior medical and ocular history were unremarkable. On examination, visual acuity (BCVA) was 20/125 in the right eye and 20/20 in the left eye, with a − 3 D myopic correction in both eyes. Intraocular pressure was 15 mmHg in both eyes. Slit lamp biomicroscopy was unremarkable. In the right eye, fundus examination showed the presence of a juxtafoveal CNV along with juxtafoveal hemorrhages nearby, and multifocal yellowish RPE changes temporally surrounding the lesion area. The fundus of the left eye was normal.

**Fig. 1:**
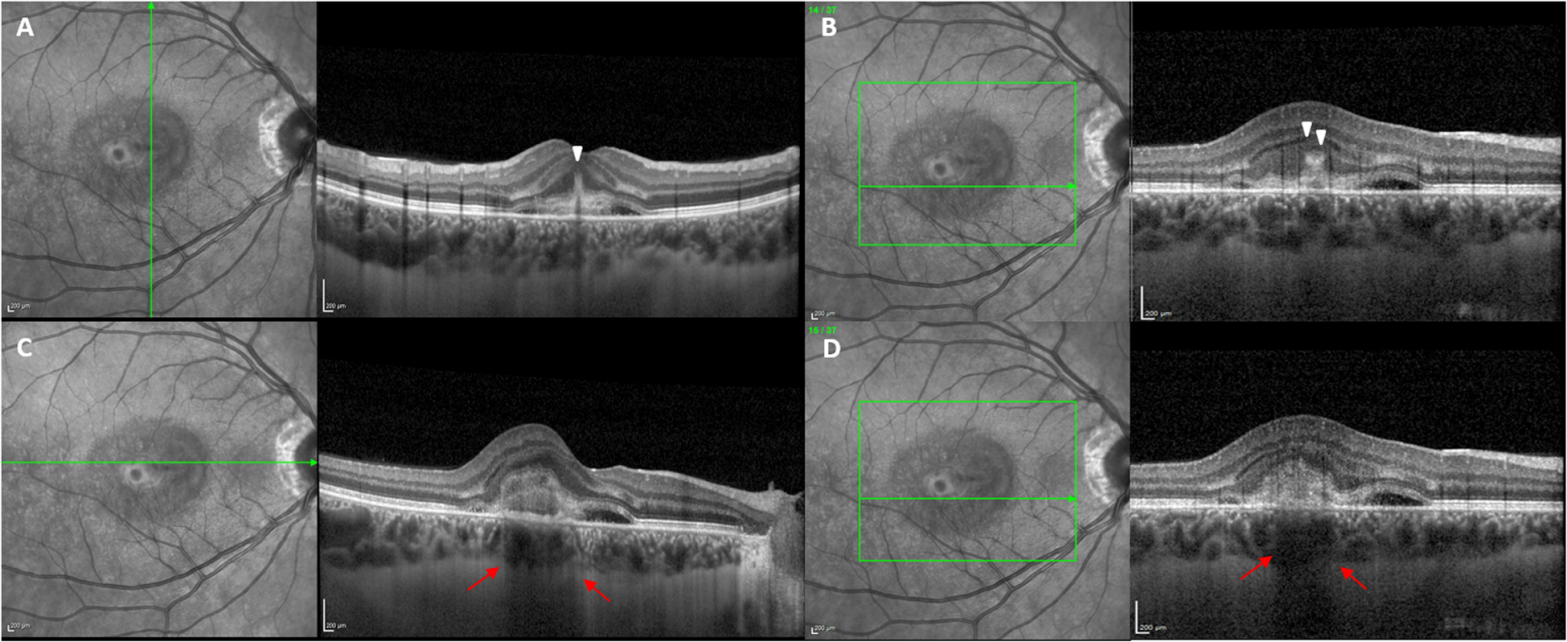
OCT scans at baseline showing type 2 CNV. **a**, **b**. White arrows show vertical finger-like projections extending from the CNV area into the outer retina, consistent with the “pitchfork sign”, typical of inflammatory CNV. **c**, **d**. Red arrows show choroidal hypo-transmission under the lesion, distinctive features for the presence of neovascularization

Optical coherence tomography (OCT - Heidelberg Engineering, Heidelberg, Germany) revealed hyper-reflective material in the subretinal space above the RPE with subretinal fluid, consistent with type 2 CNV (Fig. 1). Multiple hyper-reflective, vertical, finger-like projections extending from the area of the CNV into the outer retina were detected, resembling the tines of a pitchfork, and thus suggestive of the “pitchfork sign” (Fig. 1a,b) typical of inflammatory CNV [11]. Moreover, RPE and ellipsoid zone (EZ) disruption was detected in the site of the CNV while choroidal hypo-transmission was found under the lesion, features indicative of neovascular tissue [12] (Fig. 1c,d).

**Fig. 2:**
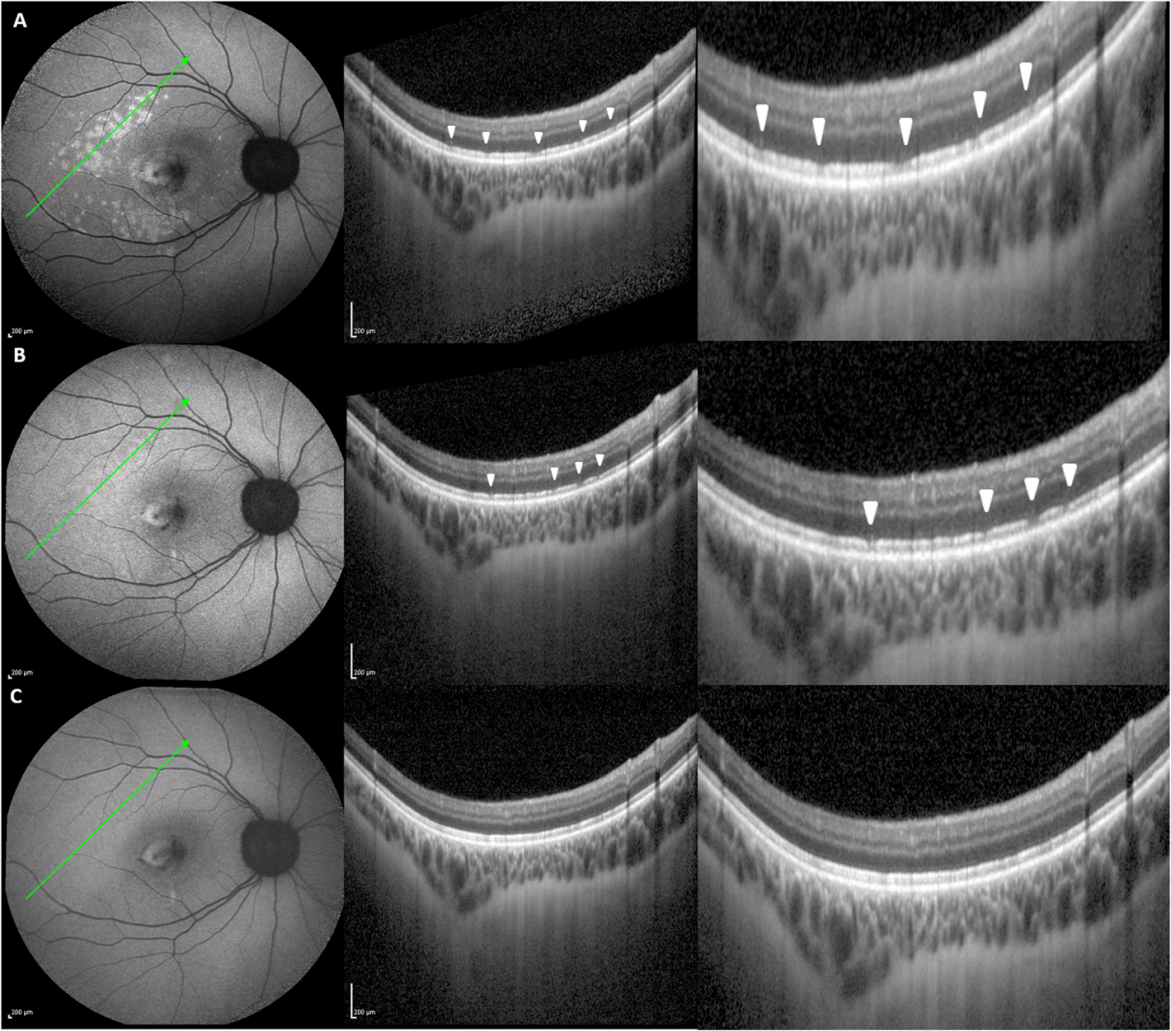
**a** Baseline. Fundus autofluorescence shows multiple hyper-autofluorescent spots surrounding the temporal side of the CNV area; OCT scans crossing these spots demonstrate disruption at the level of the outer retinal layers. **b** One-week visit post injection. Reduction of the hyper-autofluorescent spots and of the corresponding focal defects at the level of the outer retinal layers. **c** Two-week visit post-injection. Regression of the hyper-autofluorescent spots and of the corresponding tomographic focal defects

Fundus autofluorescence (FAF) showed multiple hyper-autofluorescent spots surrounding the temporal side of the CNV area, corresponding to the RPE changes detectable upon fundus examination. OCT scans across these spots demonstrated a disruption at the level of the outer retinal layers (Fig. 2a).

**Fig. 3:**
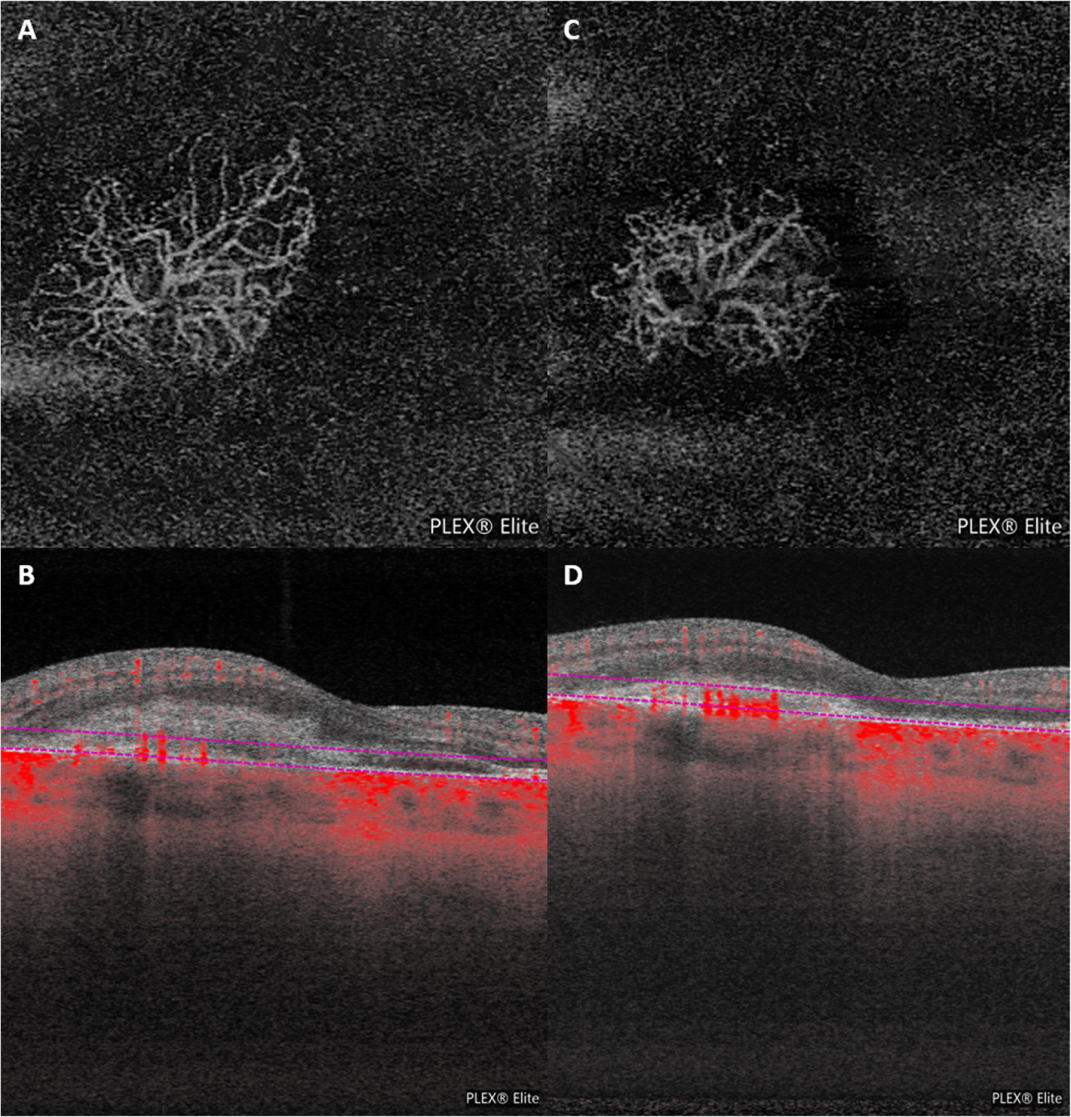
OCT angiography (OCTA) examinations. **a**, **b**. Baseline. OCTA en face scan (with corresponding structural scan) in avascular layer confirming the presence of a neovascular network **c**, **d**. Two-week visit after the injection. OCTA showing a decrease in CNV size along with a decrease in vessel caliber and branching.

The patient refused fluorescein and indocyanine green angiography because she did not want to undergo any potential risk from intravenous dye injection during pregnancy. Therefore, OCT angiography (OCTA - Zeiss Angioplex, Carl Zeiss Meditec Inc., Dublin, CA) was performed, confirming the presence of a neovascular network (Fig. 3 shows OCTA en face scan (A) with corresponding structural scan (B), confirming the presence of a neovascular network).

Based on the clinical features and multimodal imaging, the patient was diagnosed with inflammatory CNV. Multiple potential treatment options were discussed, including intravitreal injections of anti-VEGF, intravitreal injections of steroids, oral corticosteroids, or a combination thereof. The patient did not want to assume any potential risk from the use of intravitreal drugs, nor from orally administered corticosteroids. Therefore, a retrobulbar injection of triamcinolone acetonide was proposed, which was accepted by the patient after an extensive explanation of the procedure.

The patient’s eye was prepared with povidone-iodine and draped, and a topical anesthesia was applied. A 25-gauge cannula was inserted through the Tenon’s into the retrobulbar space in the infero-temporal quadrant, and 80 mg (1 ml) triamcinolone acetonide was injected (Tajoftal, Sooft, S.p.A.).

No increase in intraocular pressure (IOP) was found upon the one-day and one-week visits after the injection, nor throughout the entire ensuing follow-up (FU).

**Fig. 4:**
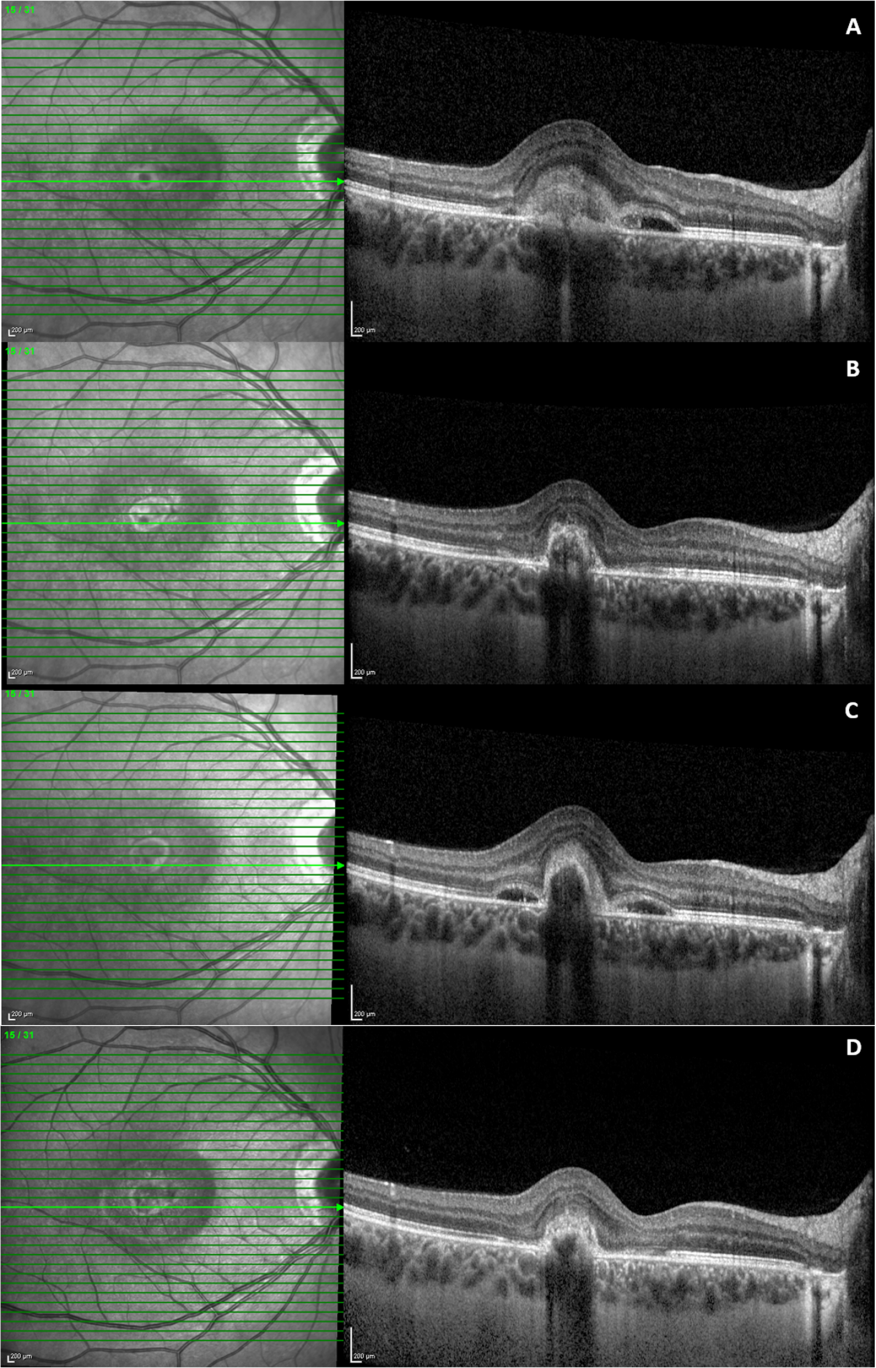
OCT changes throughout the FU period. **a** OCT scan crossing the lesion at baseline. **b** OCT performed 2 weeks after the first injection, showing subretinal fluid regression and contraction of the neovascular lesion. **c** CNV recurrence 2 months after the injection. **d** OCT performed 1 month after the second injection, demonstrating subretinal fluid regression and reduction in CNV size

At the two-week visit after the procedure, BCVA had improved to 20/32 along with a significant reduction in metamorphopsia. OCT revealed a regression in subretinal fluid and a contraction of the neovascular lesion (Fig. 4b). A reduction in the neovascular network was discovered on OCTA (Fig. 3c-d). Regression of the hyper-autofluorescent spots at the temporal side of the CNV area was detected on FAF, along with the corresponding OCT defects at the level of the outer retinal layers (Fig. 2b-c).

Two months after the injection, the patient returned complaining once again of vision loss and metamorphopsia in the same eye. OCT showed a CNV recurrence (Fig. 4c). Juxtafoveal hemorrhages were visible next to the lesion, and BCVA had decreased to 20/80. Therefore, intravitreal anti-VEGF injections were recommended. However, the patient refused intravitreal treatments, and, consequently, a second retrobulbar triamcinolone injection was performed.

One month after the second injection, OCT demonstrated both a subretinal fluid regression and CNV size reduction (Fig. 4d). BCVA in the affected eye remained stable at 20/80.

No adverse events occurred for either the mother or the baby during pregnancy and after delivery. No additional recurrences were detected until the last FU visit was performed 6 months after delivery.

## Discussion and conclusion

In pregnant women, ocular changes or an exacerbation of pre-existing retinal diseases may occur, likely as a result of hematologic, hormonal, cardiovascular, and immunologic changes. Previous studies have described a worsening of diabetic retinopathy, severe variants of central serous chorioretinopathy, and occlusive vascular disorders [4, 13, 14]. The onset of CNV during pregnancy has also been reported anecdotally [5–7, 10]. It may develop in response to a pregnancy-related increase in the activity of many angiogenic factors, which are important in maintaining placental growth, or, in patients with inflammatory CNV, it could be related to low-grade chronic intraocular inflammation, which can stimulate the release of cytokines and angiogenic factors.

In young, myopic, and pregnant women, a differential diagnosis between inflammatory, idiopathic, and myopic CNV may be challenging. In our case, multimodal imaging allowed for the diagnosis of inflammatory CNV. In particular, many typical features of an inflammatory neovascular lesion were detected by fundus examination, OCT, and autofluorescence.

On OCT, the following features were observable. First, a type 2 lesion was detected. In this type, hyper-reflective material is located above the RPE and is suggestive of neovascular proliferation in the subretinal space through a focal breach through RPE-Bruch’s membrane. Inflammatory CNV often presents as type 2 lesions. Second, the “pitchfork sign” was present. This sign is characterized by hyperreflective finger-like projections extending anteriorly from the neovascular tissue into the outer retina, and is attributed to inflammatory material such as fibrin [11]. The “pitchfork sign” has been previously described as an inflammatory CNV finding on OCT, both in inflammatory and infectious eye diseases [11, 15, 16]. Moreover, a similar finding has been reported in other settings: de Mello et al. [17] noted similar hyperreflective finger-like projections form a type 2 CNV associated with choroidal osteoma, and Vagge et al. [18] reported a similar sign in the setting of a CNV associated with vitelliform macular dystrophy. Although it is not exclusively a sign of inflammation, this feature is considered helpful in distinguishing cases of inflammatory CNV from idiopathic or myopic CNV when the diagnosis is uncertain [18]. Third, there were no tomographic signs of myopic CNV, such as a posterior staphyloma, a thin choroid, or Bruch’s membrane defects. Fourth, OCT disclosed choroidal hypo-transmission under the lesion, indicating the presence of CNV as opposed to inflammatory material alone [12]. Lastly, in our case, OCTA imaging also played a crucial role in providing a direct demonstration of the neovascular network.

Besides the abovementioned OCT features, fundus biomicroscopy and FAF also suggested inflammatory CNV. In fact, fundus examination showed focal yellowish RPE changes temporally to the lesion area corresponding to multiple hyper-autofluorescent spots on FAF examination, as those described in WDS. WDSs are considered the most frequently identified cause of inflammatory CNV [1]. Previous case reports have suggested the role of pregnancy in the exacerbation of WDS [5, 10]. As seen in our case, its typical features, consisting of multiple focal areas of inflammation at the level of the outer retina and inner choroid, may show increased autofluorescence, which could be due to a bleaching effect from the loss of the outer photoreceptors or increased fluorophores within the RPE [19, 20]. Similar to the pitchfork sign, these findings regressed after the anti-inflammatory treatment in our case.

Correctly differentiating between inflammatory or non-inflammatory CNV is crucial to allow the consideration of all possible management options. Intravitreal anti-VEGF therapy should always be considered among first-line treatments for inflammatory CNV since it blocks the primary stimulus to the new vessels’ growth. Moreover, it has been noted that inflammatory CNV may respond well to less intensive treatment regimens when compared to other CNV types, such as those secondary to age-related macular degeneration, which, on the contrary, require constant suppression of VEGF with multiple injections [2]. Oral, periocular, or intravitreal corticosteroids are further treatment options. In fact, in addition to inhibiting the pro-inflammatory factors, corticosteroids also reduce the VEGF stimulus [21, 22]. In the absence of guidelines and randomized, controlled clinical trials comparing these treatment options, it has been suggested that the best approach may be treating the neovascular component with intravitreal anti-VEGF while simultaneously controlling the inflammation with corticosteroids [2].

However, these treatment options may be limited in pregnant women, due to patient and physician concerns about the risk of systemic side effects on the mother and potential fetal harm.

Recently, the use of intravitreal anti-VEGF injections has gained wide acceptance as an off-label treatment for diseases that may affect pregnant women [6, 7, 9]. Based on a literature review, it has been concluded that while anti-VEGF treatment should be avoided during the first trimester, during the second and third trimester, it can be considered when absolutely necessary and after a comprehensive discussion on risks and benefits [8]. Similarly, the safe use of triamcinolone in pregnancy has been previously described for both sub-Tenon’s and intravitreal administration, although anecdotally [10]. Moreover, intravitreal triamcinolone has been found not to cause significant systemic serum levels [23], suggesting its use might be safe in pregnant women.

In our case, the patient refused any intravitreal or oral treatment, despite being informed on their safe use during the second and third trimesters of pregnancy [7]. Therefore, a treatment with retrobulbar triamcinolone was proposed, which had the advantage of being associated with fewer side effects when compared with intravitreal injections, including a reduced risk of steroid-induced cataract and IOP rise, and no risk of endophthalmitis and rhegmatogenous retinal detachment.

In our case, treatment efficacy in controlling both inflammation and neovascularization was demonstrated by the regression of inflammatory signs, reduction in lesion size, and functional improvement. However, the CNV relapse, occurring after 2 months, indicated a limited duration of the effect. The recurrence seemed to be less influenced by inflammation since typical inflammatory signs were no longer detectable on imaging.

In conclusion, the present study describes a rare case of inflammatory CNV in pregnancy and its management with retrobulbar triamcinolone injections. Multimodal imaging helped to demonstrate the inflammation’s origins by highlighting its typical features, therefore permitting the consideration of anti-inflammatory treatment and allowing for comprehensive patient counseling. To the best of our knowledge, this is the first reported case of inflammatory CNV in pregnancy treated with retrobulbar triamcinolone. In our case, this treatment was effective in controlling inflammation and exudation, while also stabilizing vision. No additional recurrences were detected at 6 months after the delivery and no adverse events were found for either the patient or baby. As such, this treatment may represent a suitable therapeutic option in the absence of other therapeutic possibilities.

## Data Availability

all data and material are included in the manuscript and the figures.

## Abbreviations

BCVA: Best-corrected visual acuity
CNV: Choroidal neovascularization
EZ: Ellipsoid zone
FAF: Fundus autofluorescence
FU: Follow-up
IOP: Intraocular pressure
OCT: Optical coherence tomography
OCTA: Optical coherence tomography angiography
RPE: Retinal pigment epithelium
WDS: White-dot syndromes
